# Internationalisation and moral economies in healthcare: NHS exporting and the English patient

**DOI:** 10.1186/s12992-025-01122-7

**Published:** 2025-06-13

**Authors:** Benjamin M. Hunter

**Affiliations:** 1 School of Social and Political Sciences, University of Glasgow, Glasgow, UK; 2 School of Global Studies, University of Sussex, Brighton, UK; 3 Department of International Development, King’s College London, London, UK

**Keywords:** Markets, Globalisation, NHS

## Abstract

**Background:**

Contemporary conditions require detailed study of internationalisation. This article offers a novel perspective on processes of internationalisation in healthcare, adapting an approach from higher education studies and enhancing it with insights from sociological scholarship on moral economies. The article asks how institutions and individuals respond to the globalising healthcare environment, and what this reveals about normative questions that govern healthcare provisioning in national contexts. This is pursued using qualitative data from a study on international commercial services in the English National Health Service (NHS).

**Results:**

The findings of the research demonstrate how the UK government has sought to build political consensus around specific (commodified) forms of internationalisation in a context of fiscal austerity and xenophobia surrounding the provision of public services. The English NHS has been politically re-imagined as world-leading and of interest as an export industry. Study findings show this stance is premised normatively on processes of subsidy between two apparently distinct spheres – from international (private) to national (public) – but that in practice the distinction is hazy and subsidy at times indirect, routed to individual staff members or to commercial teams. The ascendancy of this as a prevailing, politically legitimate form of internationalisation for the English NHS contrasts sharply with non-commodified alternatives decried as ‘health tourism’.

**Conclusions:**

The internationalisation framework presented in this article offers a platform for future research that can shed light on the contexts, visions, policies and contestations the emerge as healthcare institutions respond to processes of globalisation. It will be important to avoid uncritical approaches to research and policy by examining not just what forms of internationalisation find favour, and their basis in geographical and racialised hierarchies, but also how approaches to healthcare internationalisation impact inequalities within and between nations.

**Supplementary Information:**

The online version contains supplementary material available at 10.1186/s12992-025-01122-7.

## Background

Commentators frequently remark upon the ‘global’ or ‘international’ nature of healthcare. They point to cross-border flows in ideas and knowledge, service users, workers, healthcare products and technologies, and financial capital, as well as the emergence of global regulatory regimes [1–3]. Growing demand for healthcare in many settings is creating further opportunities for cross-border activity, particularly in its market-based forms [4, 5], and driving rapid expansion of a global healthcare economy. But how are these trends interpreted and pursued in a sector where, whether accurately or not, systems and services are often understood in ‘national’ terms?

This article focuses on how policy actors respond to contexts of globalisation through managed processes of internationalisation, offering two significant contributions to the study of health and other social sectors. First, it advocates closer attention to internationalisation as an understudied-concept in healthcare, the different forms that exist, and the often-implicit norms that govern internationalisation in this sector. Second, it uses the case of international commercial services in the English National Health Service (NHS) to illustrate how a specific (commodified) form of internationalisation has found favour and the justification for this. The English NHS, one of four NHS systems in the UK (the others being in Scotland, Wales and Northern Ireland), is a powerful case for studying internationalisation given its history as part of the UK’s national welfare state, its framing in explicitly ‘national’ terms, and the frequent exhortations from leading politicians that it is ‘not an international health service’ [6]. The central thesis of this article is that, in a context of fiscal austerity and xenophobia surrounding the provision of public services, the UK government has sought to build political consensus around a commodified form of internationalisation that protects free-at-the-point-of-use services for UK citizens, while decrying (non-commodified) alternatives for people deemed non-citizens.

In the next section I introduce internationalisation as an analytical concept and review the diverse, and often unconnected, ways that it has been used in healthcare scholarship. I show how it has been applied in higher education studies and the potential for cross-sectoral learning in healthcare. I then make the case for paying closer attention to the moral dimensions of internationalisation, in particular the normative basis for how internationalisation is pursued and contested, using insights from sociological studies on moral economies. Subsequent sections describe the methods for the research and then present findings on attempts to govern the national–international boundary in the English NHS. First, I set out how the English NHS has been reimagined as an export sector, entailing the commodification of access to NHS resources by the international, and its basis in relations of coloniality. Second, I show how this has been justified and understood as a process of subsidy by those involved, on the grounds that this is necessary for maintaining national public services in the context of fiscal austerity. The article concludes by offering questions to guide future research on internationalisation in healthcare.

## Internationalisation as a concept for the study of healthcare

The concept of internationalisation originates in mid-twentieth century business management literature reflecting interest in how companies expand outwards, beyond their original national context, in order to pursue sales in the international domain. But as use of the concept has spread across sectors and disciplinary fields, its interpretation has grown more varied. In the study of higher education, where use of the concept is now well-established, internationalisation is understood not only as an export strategy but as a ‘two-way street’ in which people and ideas cross borders; a broad set of activities pursued ‘to cope with globalization and to reap its benefits’ ([7], p. 291). Such activities include: representations of foreign ideas and bodies in curricula; international student exchanges and recruitment; twinning arrangements, franchising and branch campuses; Massive Open Online Courses (MOOCs), and international rankings [8, 9].

In the study of healthcare, use of internationalisation as an analytical approach has taken narrower forms than in education, resulting in a fragmented bodies of research that as a result fail to fully capture the myriad processes involved and the contextual commonalities. Existing literature is divided into three broad areas. The first follows the original interpretation of internationalisation from business management literature and examines the outward, international activities of specific healthcare providers. This includes studies on: the number of countries within which a provider operates, and the proportion of its sales and workforce based abroad [10], how hospitals have established facilities in other countries [11, 12], attempts by healthcare providers to engage cross-border users [13], and activities to combine the delivery of management consultancy services and setting up of clinics overseas with inward flows of overseas healthcare users [14]. Other studies have examined these processes without using ‘internationalisation’ as a conceptual framework (see for example [15, 16]).

A second area of scholarship discusses internationalisation in terms of foreign capital entering, and distorting, national healthcare systems. Thirty years ago, Mohan examined healthcare internationalisation as a feared ‘invasion’ by corporate healthcare from the USA was gaining ground in the UK [17]. Implicitly flipping the business management definition on its head, Mohan referred to internationalisation as the ‘growing involvement of transnational organisations in the production and delivery of health care and its associated’ ancillary’ services’ (p. 854). In applying this perspective to the UK, Mohan found multinational healthcare providers had gained a relatively small but influential foothold in UK healthcare markets. More recently, a few other scholars have used internationalisation as a concept to study similar processes in other settings. For example, Angeli and Maarse [18] drew attention to a penetration of foreign capital into the Dutch healthcare system and accompanying processes of 'cultural privatisation' and 'cultural internationalisation' that planted business management practices into the sector (p. 266). Looking at a somewhat different market, Skountridaki [19] saw the medical profession in Greece being re-made as the prospect of international income from medical travellers engendered new forms of entrepreneurialism, driving sectoral segmentation and undermining cohesion.

A third body of work discusses internationalisation in health worker education, employing an understanding of internationalisation that loosely refers to the exposure of students of medicine, nursing, midwifery and allied health professions, to foreign texts, conditions and bodies (see for example [20, 21]). Emphasis is placed on cross-border transmissions in knowledge and culture which might involve revisions to curricula, as well as opportunities for travel. One illustrative definition sees internationalisation as the process of 'purposefully integrating international, intercultural, or global dimensions into medical education in order to enhance its quality and prepare graduates for professional practice in a globalized world' ([22], p. 1694). The vision there is one of a seamless folding of the international into the national, providing cultural capital to enable workers to thrive in globalised and multicultural societies.

Each of the three areas of study outlined above differs starkly in conceptual approach, yet each offers valuable insight into the contemporary conditions of healthcare and the basis for a unifying framework. To this end, I adapt Altbach and Knight’s ‘two-way’ interpretation of internationalisation from higher education studies and apply it in the context of healthcare, understanding internationalisation in healthcare as *policies and practices adopted by institutions and individuals to respond to the globalising healthcare environment*. There are several similarities between education and healthcare sectors that provide a basis for inter-sectoral learning on the study of internationalisation, including (*inter alia*) the frequent reference to ‘national’ systems, the role of inter-personal exchanges, and the recent emergence of these sectors as frontiers for global capital. The political and economic basis for internationalisation processes is well documented in the field of education, where analyses have, for example, highlighted the importance of global trade liberalisation in creating an enabling environment for cross-border exchange, while national policy context, moves towards institutional autonomy, and the relative international standing of institutions determines the nature and direction of engagements [23–25]. Scholars note how, in higher education practice, the approach of institutions to internationalisation has been largely commercial and uncritical, tending to privilege Anglophone knowledge production, institutions of the Global North, and relations that reinforce geographical and racialised hierarchies [26].

This provides a starting point for a broader approach to thinking about the context and relations of internationalisation in healthcare, including the basis of current manifestations in turn-of-the-century (neoliberal) globalisation and the General Agreement on Trade in Services (GATS) [3, 5], and processes of corporatisation amongst public and private healthcare providers [27–29], and the antecedents in empire [30, 31]. But, to state the obvious, healthcare is not education. Amongst other differences, healthcare is characterised by a distinct collection of normative ideas that shape policy and practice, such as health and ill-health, healing and harm, and life and death. Resistance to certain processes of internationalisation has been pronounced, yielding for example the critical scholarship on entry and expansion of foreign capital into national healthcare systems (mentioned above). It is therefore important to study not just the political and economic context for internationalisation processes emerging in this sector, but also the normative factors that shape them and how they are enacted. Moral economy studies provide a theoretical foundation to do this.

### Moralising internationalisation in healthcare

Moral economy approaches have gained significant traction in social science scholarship, where their usage stems from Thompson’s and Scott’s perspective on the moral economy as a mode of economic organisation strongly determined by social norms and obligations and disrupted by the expansion of capitalism into new domains of life [32, 33]. Subsequent scholarship has sought to collapse conceptual distinctions between moral economy and market economy, arguing that social values and norms underpin all economic relations including markets. Sayer, one of the key contributors to the moral economy approach, has advocated a perspective on moral economies which studies the ‘ways in which economic activities, *in the broad sense*, are influenced by moral-political norms and sentiments, and how, conversely, those norms are compromised by economic forces’ ([34], p. 80, emphasis added). He posed a set of ‘repressed normative questions’ that govern society and that help shed light on moral economies. They include questions such as ‘Whose keeper are we? Who is our keeper?’, ‘What standards of care and provision should we expect to receive, give and fund?’, and ‘What things should not be commodified or treated as if they were commodities?’ (p. 95). Indeed, the morally informed study of what should and should not be sold is a long-standing and frequently addressed topic of analysis in the social sciences [35–37].

Boundaries and contestation around the normative questions that underpin social and economic organisation are implicit in some of the recent scholarship on moral economies in healthcare and in particular the role that markets do (or should) play. Kehr et al.’s [38] special issue is instructive, situating healthcare within the advance of ‘excessive economies’ that have become antithetical to health and that engender ‘transfigurations’ (understood as ‘radically altered understandings and transformed practices’—p. 5) in areas such as the production of hormone treatments, stem cell therapies, and patient advocacy groups. In the introduction to their special issue, the authors noted the dynamic tensions within these fields, as ‘entanglements between medicine, health, ethics, law and political economy are being (re)valued and rejected, experienced and practiced, by the involved actors on an everyday basis’ (p. 13). Kehr and others have examined how people experience, navigate and reproduce particular norms in contexts of marketisation, for example Probst on how sex workers in the EU navigate healthcare consumerism and insurance [39] and Merz et al*.* on the boundary work that takes place as healthcare providers and professionals in India and the UK grapple with shifts from philanthropic to commercial modes of collaboration [30, 31]. In other cases, the tensions result in open contestation, for example over pricing practices by pharmaceutical companies [40], or activist resistance to processes of marketisation in Spain’s healthcare system [41].

By adopting a moralised approach in the study of internationalisation, I investigate the normative basis for how boundaries between national and international are drawn in a given context: in what form(s) is internationalisation deemed legitimate, by whom, and under which conditions? Exploration of the normative dimensions of internationalisation reveals how processes are understood, justified and resisted, and the ideas deployed in the process. As I will demonstrate in the case of the English NHS, the policy preference for specific forms of internationalisation sits at the intersection of ideas around (de)commodification and entitlement to public services. In this the article points not just to *whether* services should or should not be sold, and *to whom*, but also how this is articulated through specific claims that seek to establish and guard the normative principles at stake.

## Methods

### Choice of case

My analysis of internationalisation uses the case of the public healthcare system in England—the NHS. The NHS was formed in 1948 as a key component in a post-war national welfare state, with a combination of general taxation and social insurance used to finance a mix of localised and centralised healthcare services administered chiefly through publicly owned hospitals and privately owned practices for many primary care services. That model has been largely retained but with an expansion within the NHS of private provisioning (e.g. contracting of private hospitals for surgeries) and private financing (e.g. user co-payments and private finance initiatives), while devolution of policy responsibilities to governments in Scotland, Wales and Northern Ireland in 1999 has resulted in a mosaic of NHS systems across the UK. The English NHS underwent a series of market-oriented reforms in the 2000 s and early 2010 s, making it the most commercialised of the NHS systems. In 2003, the Labour government introduced ‘Foundation Trust’ status for NHS providers, offering increased financial autonomy and borrowing capabilities, the ability to retain surplus revenue, and emphasis on imitating and competing with private organisations. This and other processes of public sector corporatisation, strengthened by the 2012 Health and Social Care Act (see later), have seen increasingly entrepreneurial tendencies within higher levels of care [42, 43], while areas of primary care including general practice and dentistry have become targets for the growth of private chains and acquisitions by US healthcare [44, 45].

The NHS, in England and the wider UK, is notable for the vigorous political commentary that surrounds it in ways distinct from other sectors and parts of the UK’s welfare state. As various commentators have noted, the very notion of ‘The NHS’ carries much affective significance for UK society as a national(ist) project and achievement [46, 47]. It is frequently anthropomorphised and lauded more for its history, values and symbolism than its present reality: a complex, constantly changing ‘behemoth’ of an institution [48]. ‘The NHS’ persistently ranks highly in polling on political concerns amongst the population of the UK and is deployed by politicians looking to garner attention and support [49]. Interest is fired up by vociferous press media coverage [50] and a community of thinktanks and commentators on hand to evaluate and propose public policy, and who function as key agents in shaping moral economies in this context.

Of particular relevance here is the way in which the *international* has been pursued and discussed in the context of an explicitly *national* health service that emerged amidst a fading British empire and benefited from its legacies. The importance to the NHS of health workers trained in other countries is increasingly well acknowledged and situated in an understanding of British imperialism [51]. Empire, the Commonwealth that followed, and the emergence of UK development aid in post-World War Two era, also provided the basis for an early period of exporting that would pivot towards countries of the Middle-East in the aftermath of oil price rises in the 1970s [52]. Post-war exports in advisory services and technologies could be subsidised by UK aid in the name of international development, with particular focus on hospital development, however these activities remained limited in scope and tailed off as under-resourced NHS providers focused their resources domestically.

The national/international boundary for service use since 2000 is particularly illustrative of how internationalisation has been shaped and contested in this context. Throughout the recent history of the English NHS, key policy reports and surrounding commentary are replete with references to the domestically oriented nature of the service: ‘serving local populations’ [53] and ‘local communities’ [54]. These categories are the intended beneficiaries of publicly funded services which are largely free and decommodified at the point of use, at least to those deemed eligible, with important exceptions including the co-payments required for certain services in relation to dentistry, optician services, and pharmacy prescriptions. The question of eligibility has proved more contestable and is frequently framed along ‘national’ lines. Criticism of the perceived exploitation of NHS resources by non-UK citizens is longstanding and dates back to the early decades of the NHS, where for example there were rumours of NHS-procured dentures found being sold privately in Baghdad ([55], p. 205). This perceived exploitation of UK public services has in recent decades been dubbed ‘health tourism’, forming part of a rhetorical repertoire of ‘benefits tourism’ that encompasses a wider range of public services [6]. Government ministers have referred with great consternation to how such activities remake the NHS as ‘*inter*national health service’ (ibid.). Yet the UK government *has* supported internationalisation, albeit preferring alternative, commodified forms that were the focus of our mixed-methods qualitative study and that I focus on in this article.

### Data collection and analysis

Three types of data are used in this article. Key policy papers produced by the UK government were retrieved from online sources and then examined for information relating to international service use, as well as a larger corpus of documentary materials produced by the UK government’s specialised unit for promoting healthcare exports—Healthcare UK. This was then supplemented with a search in Google using search terms relating to specific international actors and events, such as “Healthcare UK”. This search retrieved press media coverage and entries from the UK Parliament’s transcript archive—Hansard. Third, this was complemented with data from interviews conducted by myself or another member of the research team (see Acknowledgements) with 69 respondents in England during 2019–2023, representing NHS providers, UK government departments and arms-length bodies, private providers and consultancies, member/trade associations, royal colleges, business journalists and one university (see Table 1). Respondents were identified from institutional websites and contacted by email. During interviews they were asked questions relating to the nature and purpose of international commercial services, and the practices deemed (il)legitimate.

**Table 1:** Summary of interview participant details

Key Current/Former Affiliation	Number of Respondents
NHS provider (including subsidiary companies)	**30**
UK Government department/arms-length body	**13**
Private provider/consultancy	**9**
Member/trade association or network	**8**
Royal College	**5**
Business journalist	**3**
University	**1**

I coded the resources line-by-line with assistance from Nvivo, assigning an initial set of descriptive codes derived from the moral economies perspective outlined above and which related to the (contested) notions of legitimate practice across several areas of international commercial practice and the key actors involved in constructing these (see Supplementary materials for a table of codes). During this process, I observed that NHS providers were engaging in internationalisation processes similar to those undertaken in higher education, and that they experienced, or were subject to, significant tensions between domestic responsibilities and international interests. A second phase of analysis focused on these tensions and grouped codes into two categories: one charting the normalisation in UK policy of NHS exporting; the second on the process of subsidy that underpins this and how this process operates in practice.

### Research ethics

Ethics approval for the project was granted by King's College London. Respondents were informed about their rights to anonymity and confidentiality, and the timeframe for withdrawing participation, at the beginning of the interview. They gave oral consent for participation. Due to potential issues of commercial and political sensitivity, interviews were not recorded and the interviewer(s) took detailed notes, including some short quotations, during and after the interview. Two respondents requested to see the notes afterwards and one made additions to the text to add further detail. All notes were anonymised prior to archiving.

## Results

### The policy context favouring commodified internationalisation

#### Reimagining the english NHS as export sector

The recent period of NHS exporting, beginning in the mid-late 2000 s, can be distinguished from earlier rounds of international commercial work by its global ambition and entrepreneurial character, coming as it did soon after the introduction of a corporate-like ‘Foundation Trust’ status for NHS providers. There was an increasingly celebratory tone of political commentary around the NHS in the late 2000 s, a marked change from more pessimistic assessments of the late-1990 s and early 2000 s. The 2007 publication of *Global Health Partnerships: The UK Contribution to Health in Developing Countries* by former NHS Chief Executive Nigel Crisp, at the asking of Prime Minister Tony Blair and Secretaries of State for Health and for International Development, saw Tony Blair’s foreword describing the NHS as possessing ‘skills and experience that other countries could learn from’ [56]. The following year, to celebrate the 60 th anniversary of the founding of the NHS, the Nuffield Trust compiled a set of testimonies – *Rejuvenate or Retire: Views of the NHS at 60* – which included frequent reference to the NHS as a global trend-setter and source of innovation [57].

A political and economic crisis provided the context for celebrations of the NHS to spill over into a push towards international markets. In March 2010, in the aftermath of the Global Financial Crisis and with fiscal austerity looming, the NHS announced plans to create NHS Global within the Department of Health—a centralised unit with the aim of encouraging greater provision of commercial services by NHS organisations in other countries. Secretary of State for Health Andy Burnham highlighted the perceived success of BBC Worldwide as a model for commercialising and exporting UK expertise, noting the opportunity for NHS providers to follow the same path. Burnham’s plan was an extension of the work already being done by an ‘International’ team within the Department of Health and who provided support for UK companies (not just NHS providers) to expand their international business ([56], p. 7). But within months, Burnham’s Labour counterparts had lost a general election, to be replaced by a Conservative-led coalition government.

The idea of an entrepreneurial, exporting NHS played to the politics of the new government, and was remarked upon in the Treasury's 2011 Plan for Growth, which noted that a ‘proactive, entrepreneurial NHS Global would ‘make the most of the NHS brand internationally’ ([58], p. 97). However, NHS Global was coming under pressure due to its failure to secure any contracts [59] and, in a moment revealing of rival moral visions for NHS internationalisation, NHS Global was moved out of the Department of Health and into the UK government’s trade agency, UKTI, where it became Healthcare UK. The Department of Health had harboured some interest in a wider mandate encompassing philanthropic and commercial international activities ([60], p. 12), but was outflanked by other agencies who wanted to re-package NHS Global with an exclusively commercial mandate. Study respondents involved in this process suggested that NHS Global succeeded in promoting the idea of NHS exporting and made some initial in-roads in Saudi Arabia, but that the decision to move it to UKTI reflected hopes that the latter would be better placed to identify and pass on business leads using its network of trade representatives in UK embassies and consulates around the world.

After two years of what had been limited progress as NHS Global, two key developments in 2012 provided impetus to launch Healthcare UK and with it the UK government’s operationalisation of a market-oriented approach to NHS internationalisation. First, the Health and Social Care Act, spear-headed by Secretary of State for Health Andrew Lansley, was adopted in 2012 and encouraged NHS Foundation Trusts to pursue private income generation (domestically and internationally) by lifting a cap on private income; it was increased to 49% of income. With the restrictions lifted, hospital management had greater freedom to pursue opportunities for commercial revenue generation, and Healthcare UK could provide a mechanism to support this. Second, in July 2012 the NHS and Great Ormond Street Children’s Hospital featured prominently in the Opening Ceremony of the London Summer Olympics, to great domestic and global acclaim. Within weeks, Parliamentary Under Secretary of State for Health Anne Milton was publicly championing the idea of Healthcare UK in a radio interview. Healthcare UK’s first annual report, published in 2014, cited the Olympic ceremony and ‘the potent image of the NHS logo’ as stimulating ‘considerable interest in what the UK might have to offer’ ([61], p. 4), though some providers benefited more than others—one of our NHS provider respondents remarked that Great Ormond Street was able to use the publicity to distinguish itself “from the rest of the pack”.

#### Selling universalism

Healthcare UK was launched at the 2013 Arab Health trade show in Dubai, by Parliamentary Under-Secretary of State for Health Frederick Curzon (officially titled Earl Howe) and surgeon and UK Business Ambassador, Ara Darzi. Its initial objectives were to engage providers, raise the international profile of UK healthcare, identify business opportunities, and convert these ‘leads’ into ‘wins’ (i.e. signed agreements), through a combination of trade missions, summits and meetings trade missions, summits and meetings [62]. The choice of initial ‘priority [geographical] markets’ (listed in the 2015 annual report as China, Hong Kong, Brazil, Turkey, India, Saudi Arabia, UAE, Kuwait, Oman and Algeria) represented an eclectic mix of large middle-income countries who were increasingly been seen by UK leaders as rival powers in a multipolar global economy, and former British Colonies/Protectorates ([63], p. 2). Materials produced for a 2014 forum with businesses provided further insight, listing ‘favourable inter-governmental relationship’ and ‘UKTI active in market’, signs of historical British presence, as factors useful for determining target countries ([64], p. 37). These countries were reported to perceive the NHS as ‘cutting edge in many areas of healthcare delivery’ and were ‘keen to learn about the UK’s approach to delivering universal healthcare’, reinforcing the celebratory ideas of the Crisp report and others ([63], p. 3).

In 2015, Healthcare UK was re-launched in Peru, where the national government was launching an infrastructure public–private partnership programme akin to the NHS’ private finance initiatives. Managers sought to relive the euphoria of the 2012 Olympic Opening Ceremony by organising a re-enactment of the scenes involving the NHS. By the time of its relaunch, Healthcare UK was reporting it had identified business ‘leads’ of £9.2 billion and business ‘wins’ of £3.7 billion, though the values specific to NHS providers were not provided [65]. Respondents with experience working with Healthcare UK were keen to emphasise its focus on expanding the commercial activities of NHS providers *beyond* the treatment of users within the UK, and promotional materials point to examples such as private hospitals and clinics established and/or managed in United Arab Emirates by Moorfields, King’s College Hospital and South London and Maudsley; and hospital development contracts in India and China for King’s, The Christie and Northumbria. Meanwhile Healthcare UK was pitching the NHS as a system that could be packaged and sold internationally, making frequent reference to UK performance in the Commonwealth Fund’s rankings, albeit tending to omit mention that the ranking includes only 11 countries:

The NHS is admired around the globe and is widely seen as an exemplar of universal health coverage, being ranked consistently as the world’s best healthcare system by US-based think tank the Commonwealth Fund. It is the third time in a row that the study, which is undertaken every three years, has found the UK to have the highest-rated health system. ([66], p. 6)

References to the Commonwealth Fund rankings, and more generally to the NHS as an exemplar for universalism, sat uneasily with a reality in which agreements being signed internationally were often to develop private services with for-profit corporate partners such as Chinese conglomerate Rongqiao (Northumbria), UK investment fund manager Ashmore (King’s College), and Middle-East provider MACANI (Maudsley). When Healthcare UK launched its NHS Export Collaborative in 2021, the aim was to encourage closer collaboration between NHS providers with a view to them competing with US multinationals to win larger international contracts of the kind unlikely to involve the creation of publicly financed and provided healthcare. Indeed, the first success of the Collaborative was the formation of a King’s-led consortium which respondents said was a product of NHS England Chair David Prior’s brokering of a meeting to create consortia amongst ten leading NHS providers. King’s has a track record working with other NHS providers to develop large private for-profit facilities in UAE, Saudi Arabia and Nigeria, indicating the likely direction of work for the new consortium.

Senior politicians have played a key role in guarding this vision for NHS internationalisation, championing commodified models while rejecting and vilifying non-commodified forms dubbed ‘health tourism’. Leading Conservative politician and general practitioner Liam Fox, who as UK Secretary of State for Trade and Industry (2016–2018) could be found travelling the world to promote the commercial potential of the NHS [67], has been a leading critic of so-called health tourism, once likening the NHS to a Disneyland for health tourists [68]. Health Secretary Jeremy Hunt, one of various politicians who decried the prospect of an ‘international health service’ [69], could be found lending his support to the Indo-UK Institutes of Health—plans for 11 private hospitals to be built across India and branded by leading NHS providers: ‘I am proud that our NHS will be used as an example of gold standard healthcare in India – it is only right that our world-leading knowledge and expertise is shared across the globe’ [70], ‘shared’ in this case being euphemism for ‘sold’. There is no small irony to NHS providers selling services in a country which has not only subsidised UK healthcare through extensive migration of health workers in the contemporary era [30, 31], but which through colonial extractions provided funds for the very foundations of the UK welfare state [71].

The concern with health tourism appears to have not been so much that services have been provided to international users; rather that they have not been provided in forms that are suitably commodified. Indeed, initiatives such as the 2014 NHS Visitor and Migrant Cost Recovery Programme have sought to remedy exactly that—to extend and enforce charges for healthcare for non-UK nationals, thereby bringing services into line with a more politically palatable, commodified approach. This points to an underlying normative question around how to resource services in the English NHS and two principles that govern national–international boundaries in this regard: first, that profit made on (international) commercial income can and should be used to subsidise public services in England, and second, that public resources should not be used to subsidise (international) commercial services. These are discussed in turn below.

### The normative basis for commodified internationalisation

#### Principle 1: ‘More money for the NHS’

For decades, NHS provider budgets have been squeezed by growing demand for services and rising costs, intensifying in the past 15 years due to fiscal austerity and real terms public funding settlements that, when adjusted for changes in population, led to stagnating funding including cuts in some years [72]. As a result, there has been intense political interest in finding new avenues for revenue generation that can supplement public funding, with (primarily domestic) private sources of income seen as one option. NHS Foundation Trusts, introduced in 2003, would be corporate-like in their operations and management, arousing several concerns, not least the potential to become oriented towards private rather than public service provision. Members of the ruling Labour party instigated the introduction of a cap on private patient income generation for Foundation Trusts, fixed at the proportion of a hospital’s total income derived from private patients in the 2002/03 financial year [73]: the average cap across England was 2%, but was higher in some London hospitals already delivering significant volumes of private services. Such was the concern amongst some providers (for example Great Ormond Street Hospital) that this would impede the growth of private income streams that they delayed attaining Foundation Trust status [74]. As a result of the cap, annual income for NHS providers from ‘non-NHS overseas patients’ rose relatively slowly during the 2000 s, from £9 million in 2003 to £13 million by 2012 [75, 76].

As noted in the previous section, it was not until the late-2000 s and into the 2010 s that political and economic space opened for the UK government to encourage NHS exporting, at a time when UK state-citizen relations were being remade by deepening fiscal austerity and welfare state retrenchment. In discussing the motivations of their organisations to export, study respondents pointed to growing budget deficits amongst their employers and the dire state of facilities needing renovation; as one international commercial lead at a provider in London noted, “we all have bills to pay”. For a respondent from a provider considered more elite, it was not about obtaining funds to maintain a basic level of service, but about generating the resources through which to stand out internationally: “NHS funding doesn’t make you a world-class hospital” (international commercial lead, NHS provider in London).

Agencies of the UK government have, unsurprisingly, avoided referring explicitly to public spending cuts, and instead focused on benefits to the NHS in more abstract terms. When championing proposals for NHS Global in 2010, health secretary Andy Burnham highlighted the opportunity to ‘exploit the commercial potential of the NHS on the world stage, bring benefits back to patients here in the UK, and help the NHS fulfil its humanitarian role in world health care’ [77]. Three years later, it was the turn of health minister Frederick Curzon to proclaim the benefits to NHS services: ‘Healthcare UK is good news for the UK economy which will benefit from the extra jobs and revenue created by our highly successful healthcare industries as they trade more across the globe. It also means more money for the NHS across the UK’ [78]. By 2016, Healthcare UK had adopted a ‘five Rs’ case for promoting NHS exports: revenue, reputation, research, recruitment and retention, and reach [79], though our respondents working in NHS providers emphasised the prominence of *revenue* over other factors in the ‘five Rs’; “surely it [more revenue] has to be a good thing?” (international commercial lead, NHS provider outside London).

The premise is that profit from international commercial income can and will be ‘reinvested’ into NHS public services. Our respondents in commercial teams reported various models for this: some reimbursed clinical departments with large payments that would more than cover a locum worker for their clinicians’ time; others did so in-kind, by providing funds for education and training rather than staffing. Another model saw profits from international income going to the provider’s general budgets where they would help to increase the overall budget available for the provider, or at least reduce a budget deficit. However respondents in commercial teams were at times sceptical of the benefit of this latter process, with one referring to their London-based employer’s budget deficit as a “black hole” into which financial surplus from the commercial team disappeared: “what’s a £50,000 surplus if the deficit is [several] million?”.

Yet in some cases, the process of reinvestment seemed more indirect, routed through individual staff members or the commercial team. Respondents reported that where they considered they were using the private consulting time of clinicians, they would reimburse the clinician personally instead of the clinical department; the idea was that this does not weaken public service provision, but this logic seems questionable in a context of growing reliance amongst NHS services on the contracting of private providers. Commercial leads wanted to use at least some of the profit from their contract income to ‘reinvest’ in the growth of their commercial team, offering what they saw as a virtuous spiral in which expanding their team could secure more and larger contracts, generate more income for their employer, and in turn lead to more funds to grow the team further. One respondent was particularly aggrieved that funds generated through a large international contract were absorbed into general budgets rather than being used to expand their team’s work. The use of subsidiary companies, for example in the cases of King’s College Hospital and Guy’s and St Thomas’, appears key to enabling commercial teams to retain and reinvest funds in their own work, with a respondent at a different provider noting that working through a subsidiary seemed a necessary strategy to “grow your business” beyond relatively small-scale activities. This form of ‘reinvestment’ does however raise questions regarding the extent to which public services end up benefitting from a growing commercial presence in the provider, particularly in a context where commentators have noted an absence of evidence to support claims of cross-subsidy between private and public services [80].

#### Principle 2: Protect ‘English patients’

Despite substantial enthusiasm from certain quarters regarding the financial potential of NHS international commercial services, there have been lingering concerns regarding the risk to public service provision. When the launch of Healthcare UK was announced, it attracted critical comment that its activities could undermine domestic services, with a representative from The Patients Association stating “we’re all for hospitals branching out and making money elsewhere providing it doesn’t affect their patients [in the UK]” [81]. Andy Burnham, by then a shadow health minister, invoked the idea of “English patients” who would lose out to “patients from overseas” as Burnham sought to distinguish between Healthcare UK and his own plans for NHS Global two years prior:

- I want to see other countries borrowing our ideas, our expertise, and that’s what our plans for NHS Global were very much about. I am worried that this is something very different altogether. This, to me, sounds like a hard-nosed commercial plan to fill NHS beds with private patients. […] The government have brought in the provision for the half-privatisation of English hospitals. They are allowing hospitals to make up to 49% of their income from the treatment of private patients. This proposal today very much has to be seen in that context because there is massively increased scope here for English hospitals to bring in patients from overseas, put them into beds here, and that of course will have a detrimental impact on waiting times for English patients [82].

Perhaps mindful of such criticisms, proponents of NHS exporting have fostered the idea of a separation of role and resources, between domestic (public) and international (private) provision. Health minister Anne Milton, commenting on the proposed Healthcare UK in 2012 claimed that any up-front investment needed for overseas work would come from existing streams of private patient income, not NHS public resources; she cited Moorfields Eye Hospital Dubai as an apparent success story in that regard [83]. The informality and messiness of some of the reinvestment models described in the previous section makes it hard to know whether such a firewall between public and private funds does indeed exist, particularly for providers who do not use subsidiaries for their international commercial work. NHS providers are not required to publish data for their spending on international commercial work therefore it is impossible to compare to the private patient income that they are obliged to report.

The line between public and private resources seems particularly blurred in the case of staffing. Respondents sought to justify their use of clinicians’ time for international commercial work by pointing to the payments made to individuals and to clinical departments, the wider benefits of international travel opportunities for staff morale, and the supportive response from some heads of clinical departments. Some emphasised that they tried to minimise use of clinicians’ time by “rolling up their [own] sleeves” (international commercial lead, NHS provider outside London), so as not to take clinicians “away from the frontline” of NHS practice (international commercial lead, NHS provider in London). Yet, ultimately, they required senior specialists for certain tasks and acknowledged there are only a finite number of available and that their experience and skills are difficult to replace with locum cover; as one London-based respondent noted, it is not easy to “just buy in [a consultant] for the day”. Indeed, respondents also highlighted instances of resistance they met when trying to find clinicians to deliver a package of work amidst increasingly strained domestic clinical services and staffing shortages that have been intensified by the COVID-19 pandemic.

Somewhat counterintuitively, senior NHS leaders have sought to deploy recent challenges facing NHS providers as justification for doing *more* exporting: in 2018, outgoing Chair of NHS England Malcolm Grant argued that ‘NHS trusts not only have the capability, but also responsibility to develop strategies to export their expertise and services, *even – and arguably especially – when they are under severe pressures in maintaining vital services at home*’ ([84], emphasis added). Some respondents and promotional materials even saw COVID-19 as a business opportunity in which NHS and other providers could export its ‘expertise and insight gained through the effective deployment of resources coupled with successful use of data and analytics’ [85], p. 2). How this enthusiasm could be squared with widespread staffing shortages remains unclear, but the adage “never let a good crisis go to waste” appears particularly salient for some of these champions of NHS exporting.

## Discussion and conclusions

There has been widespread recognition of the global character of healthcare, and significant attention to processes of transnationalisation and globalisation [1, 86–88], yet the concept of internationalisation has been under-utilised in the field to date. This is particularly noticeable when compared to the study of higher education. Internationalisation offers a perspective on the policies and practices adopted by institutions and institutions in national contexts as they seek to manage interactions with the increasingly globalised healthcare environment. It provides a basis through which to consider the contexts that drive and shape international engagements, and how they are understood, as well as revealing tendencies towards commercialism and the reproduction of geographic and racialised hierarchies. There is therefore significant scope for further study on internationalisation in healthcare systems, and possible questions for future research include: What is the context and vision for healthcare internationalisation in different settings? How does it interplay with processes of coloniality and of corporatisation in public and private sectors? How are differing visions contested, and which forms find favour? What is the impact of internationalisation on health, healthcare and inequality, both in one country’s healthcare system and in others affected by extension? Ultimately, who benefits?

Using a moralised approach to study the international provision of commercial services in the English NHS, I have shown how the UK government has sought to build political consensus around one commodified form of internationalisation in a particular political and economic context. The development of NHS exporting during the past 15 years has been encouraged by fiscal austerity and market-oriented policy reforms that reimagined the English NHS as world-leading and of economic interest for exporting, emphasising the revenue possibilities. Meanwhile, in a political context of intensifying xenophobia best illustrated by the UK government’s ‘Hostile Environment’ policy, an alternative, non-commodified form of internationalisation was constructed and decried as ‘health tourism’. To date, these two areas of policy have been studied in isolation, as reflecting relations of entrepreneurialism [42, 43] and of nationalism [6, 51], respectively. My approach considers them as attempts to govern the national-international boundary, and its intersections with public-private/commodified-decommodified zones of provision, and the (il)legitimate forms of internationalisation that result. Indeed, access to services is just one emerging area of internationalisation, and an understanding of the contemporary nature of the English NHS requires closer attention to the ebb and flow of multiple internationalisations, and of their impact on health outcomes and health equity in England and in other settings, something which was not possible in this article.

My analysis in this article has paid particular attention to the norms that underpin international commercial services in the English NHS. In this my work reflects recent scholarly interest in making explicit the norms that govern healthcare markets [30, 31, 38–41], extending that line of research to consider how policy actors seek to govern engagements with international markets in a sector where systems and services are often still understood in ‘national’ terms. In Ondaatje’s classic novel of the same name, the 'English patient' symbolises the construction and rejection of national identity in a context of fading European empire. Andy Burnham’s invocation of the English patient in 2012 asserted national identity to govern welfare entitlements in the context of further economic decline for a colonial power: Burnham’s English patient lays claim to a taxation-funded free-at-the-point-of-use NHS, while patients deemed non-national face an alternative, fee-based system for financing. Proponents of internationalisation in its commodified forms take this further, offering to create a distinct system in which the NHS is commercial exporter and generates cross-subsidies for public services in austerity England. As studies of empire begin to trace how expropriations from those deemed non-national were used to construct twentieth century welfare states [71], it is striking that the UK government should now fall back on similar processes to preserve what remains.

The interview data indicate that in practice the separation between systems – national/international and public/private – is hazy and that the claimed reinvestments into public services involve processes that can seem very indirect. The limited publicly available information on spending and revenue generation in this area makes any net financial benefit impossible to discern. In this the findings mirror wider suspicion towards claims of cross-subsidy from private to public services in the English NHS [80], and provide further weight to qualitative evidence of the potential for reverse cross-subsidy from public to private [89]. Nonetheless, the trend towards these activities seems likely to persist amidst continued resource shortages in the English NHS, the high-level support for exporting, and a propensity for market-oriented institutional change to become self-perpetuating. Experiences with internationalisation in higher education indicate a tendency towards commodified models of provision that in turn recalibrate, remake and reconstitute institutions, cultivating further intensification of commercial activities [90], and other scholars have pointed to what seems to be similar systemic changes playing out amongst NHS providers [27]. Contrary to the proclamations of certain politicians, England *has* an international health service, but it is unclear whose interests it serves.

## Supplementary Information

Supplementary Material 1.

## Data Availability

The dataset supporting the conclusions of this article is available in the UK Data Service repository, https://reshare.ukdataservice.ac.uk/856673/.
