# Supplementary material for: Internationalisation and moral economies in healthcare: NHS exporting and the English patient
Source: Global Health. 2025 Jun 13;21:37. doi: 10.1186/s12992-025-01122-7 (PMC12166583; doi:10.1186/s12992-025-01122-7)
Supplement: Supplementary file 1 — Supplementary Material 1. [file 12992_2025_1122_MOESM1_ESM.docx]

| Category | Parent code | Child code |
| --- | --- | --- |
| What is considered legitimate practice? How do actors legitimise their practices? And how are these notions challenged or subverted? | - Justifications - 'exporters' | - Financial benefit to exporter - Improve healthcare in exporting country - Improve relations with nearby organisations - International reputations - Jobs and tax revenue in exporting country - Opportunities for clinical research and extraction of data - Reputational benefit to exporting organisations - 'Secondary benefits' - Soft power and health diplomacy - Workforce benefits for exporters |
|  | - Justifications - 'importers' | - Belt and Road - Can offer referrals abroad - Competition provides choice for consumers - Financial benefit - Improve healthcare quality and standards - Makes development projects more attractive to home buyers - Online training increases reach - Opportunities for future migration - Reputational benefits - Social development - Technological acquisition - Workforce benefits for importers |
|  | - (Un)acceptable attitudes and working practices | - Buyer-seller relations - Internal logics and practices - Market organising - who can participate |
|  | - NHS and its partners' identities (or 'brand') | - Confusion around structure of NHS |
